# Reliability-based assessment of road design features and crash risk using a socio-economic index for safety prioritization

**DOI:** 10.1038/s41598-026-36005-3

**Published:** 2026-01-29

**Authors:** Hossein Saedi, Ali Abdi Kordani, Hamid Reza Behnood

**Affiliations:** Department of Civil Engineering– Transportation Planning, Faculty of Technical and Engineering, Imam Khomeini International University (IKIU), Qazvin, Iran

**Keywords:** Highway safety, Crash risk, Reliability analysis, Prioritization, Engineering, Mathematics and computing, Risk factors

## Abstract

This study investigates the relationship between road design features and crash risk on a 186 km segment of Highway No. 36 in Iran. A socio-economic risk index was developed by integrating Empirical Bayes crash predictions, severity-based social crash costs, and construction cost estimates. This index was incorporated into a reliability framework, where limit-state functions and Monte Carlo simulation were used to compute the exceedance probability of crash risk. Geometric data were collected through field surveys, and traffic and crash data were obtained from the Khorasan Razavi Road Maintenance and transportation organization’s database (2019–2023). The results show that horizontal curves have the highest crash risk, while segments longer than 4 km exhibit the lowest values. Crash risk also increases with wider lanes, gravel shoulders, greater shoulder widths, and embankment slopes steeper than 4%. Grades between 0 and 3% reduce risk, whereas steeper grades elevate it. Guardrails demonstrate mixed effects, reducing risk at lower levels but not consistently at higher ones. The reliability-based probabilistic framework integrates crash data, societal costs with severity, and construction costs to systematically prioritize safety interventions, offering a clear methodological advantage over deterministic approaches.

## Introduction

Highway safety represents one of the most critical dimensions of road transport systems^1^. Assessing crash risk helps identify high-risk zones and prioritize safety improvement initiatives^2^. By applying risk assessment models, road segments with the highest likelihood of crashes can be detected, which enables the implementation of targeted and effective preventive measures^3,4^. Moreover, risk-based prioritization of safety projects ensures the optimal allocation of limited resources, resulting in a significant reduction in the societal costs associated with highway crashes^5,6^.

Reliability analysis and risk indices based on the social costs of crashes have become important tools for supporting decision-making in highway safety management^7,8^. These approaches not only assist in identifying existing safety concerns but also provide mechanisms for assessing the effectiveness of corrective interventions^8,9^. Additionally, the use of crash prediction models allows for the simulation of high-risk areas, which aids in refining safety strategies and ensuring optimal project planning^10^. Together, these methodologies strengthen safety-related decision-making and contribute to reducing transportation risks^11^.

Most crash risk models analyze frequency or cost separately and lack a framework integrating economic, social, and probabilistic factors. Moreover, the uncertainty associated with exceeding acceptable risk thresholds is rarely assessed in a formal reliability context.

To bridge these gaps, the present study introduces an integrated framework that simultaneously incorporates geometric design parameters and uncertainty into crash risk assessment. The framework is applied to a 186-kilometer section of Highway No. 36 in Iran, between Shadmehr and Sabzevar, using geometric, traffic, and crash data collected between 2019 and 2023.

The first step of this study involved modeling the social costs of crashes incorporating the Emperical Bayes method recommended by Highway Safety Manual (HSM), as well as the severity-based social cost values. Subsequently, a risk index was calculated by comparing the predicted social losses to the construction costs of undivided two-lane highways, utilizing data from the Florida Department of Transportation (FDOT) project^12^. In this study, two U.S.-based monetary parameters were incorporated to ensure internal consistency in the formulation of the risk index. The average construction cost for undivided two-lane rural roads was sourced from the FDOT, reported as $5,549,319.13 per kilometer as of October 2024. In contrast, the social crash cost values—used to quantify crash consequences—were adopted from the HSM recommendations. Both cost components rely on U.S. data and are applied consistently to maintain coherence. Furthermore, since the primary goal of this research is to develop a methodological framework, the resulting risk indices maintain relative validity even when applied to Iranian road segments. This risk index was computed for all segments of the highway, and reliability analysis was performed to estimate the probability of exceeding various risk threshold values. Finally, diagrams were generated to analyze risk based on design features. These results were used to develop a framework for prioritizing safety improvement projects, enabling more effective management of highway safety and resource allocation toward the highest-risk projects.

Few studies systematically explore how highway design features affect crash risk across diverse traffic and geographic contexts. Most studies examine individual road geometries, such as curves or slopes, but rarely consider all variables shaping complex transport systems^4,9^. Many studies examined crash risk with geometric or traffic variables, but few integrated socio-economic factors probabilistically. As such, there remains a clear need for a comprehensive model that simultaneously incorporates highway design, traffic features, and crash statistics into an integrated reliability-based approach to crash risk.

The primary innovation of this study lies in the development of a combined risk index model that simultaneously incorporates both social and economic data. Most studies analyzed crashes or cost–benefit separately^11,13^, but this research integrates social crash and highway construction costs. This comprehensive approach provides a more holistic understanding of the socioeconomic dimensions of crash risk assessment. This integration allows the proposed risk index to reflect not only the societal burden of crashes but also the economic trade-offs associated with infrastructure investment. By comparing predicted crash losses to construction costs, the model provides a rational basis for prioritizing safety improvements where the potential social benefits of risk reduction outweigh the required financial investment. Furthermore, the application of reliability analysis to estimate the probability of crash risks exceeding design thresholds represents a methodological advancement that has not been extensively investigated in prior research^3,6^.

Beyond these theoretical contributions, this study utilizes accurate, real-world data and field-based methods to investigate the complex relationships between highway geometric characteristics, such as shoulder width, shoulder type, embankment slope, and curvature, and crash risk. Whereas previous research^14,15^ typically examined a limited set of variables, this study conducts a comprehensive, simultaneous analysis of multiple geometric and environmental factors. Moreover, by establishing a framework for prioritizing safety improvement projects based on quantified risk assessments, this research makes a substantial contribution to advancing highway safety policy. This area has not been adequately addressed in previous large-scale, multi-criteria studies^5,9^ .

Jalayer and Zhou^16^ focused on reliability analysis for roadside features on two-lane rural highways, finding that obstacles and steep embankments notably increase crash risk. This study illustrated the necessity of considering roadside environmental conditions in addition to geometric design to mitigate crash occurrences. In a related study, Dhahir and Hassan^17^ applied reliability analysis to naturalistic driving data to assess the impact of geometric design factors like curve radius and shoulder width on crash risk. They found that larger radii and properly designed shoulders significantly reduce lane departure risks, underscoring the importance of reliability in highway design. Similarly, Shalkamy and El-Basyouny^18^ used multivariate models to analyze the relationship between collision risk and reliability outcomes for horizontal curves. Their results show geometric and environmental factors, like curve radius and surface, affect crash risk and support reliability-based models. The study by Himes and Donnell^19^ examined the Safety Effects of the Horizontal Curve Reliability Index. Their research demonstrated that this reliability index can accurately predict collision risks, thus providing valuable guidance for highway designers aiming to improve safety through optimized geometric features. Shalkamy et al.^20^ explored the impact of geometric features of horizontal curves on crash risk, identifying that larger curve radii and proper superelevation significantly reduce crash risks. The study also emphasized the role of reliability measures in curve design to predict and mitigate crashes, offering insights for prioritizing highway safety projects. The study by Alsaleh et al.^21^ introduced a reliability-based optimization framework for the geometric design of horizontal curves, integrating design consistency with crash risk analysis. The results showed that optimizing curve design could significantly reduce crash risks, highlighting the potential of probabilistic models to enhance highway safety under varying conditions. Finally, the study by Prencipe et al.^22^ examined injury severity in shared micromobility crashes. Their findings highlighted the importance of infrastructure characteristics, traffic volume, and shared mobility patterns in shaping crash outcomes. This study is relevant as it emphasizes the need for targeted interventions in transportation networks to reduce crash risks and injury severity.

## Methods

Reliability methods have been developed over the past three decades, making them particularly well-suited for hazard analysis (e.g^18,23,24^. , . To adapt reliability methods for hazard analysis, The present study formulates the limit-state function in relation to crash risk indicators, incorporating the probability of exceeding defined thresholds for safety performance. In this approach, the limit-state function, *G(x)*, represents the event where the intensity *R* exceeds a specified threshold $\:{R}_{0}$. Therefore, the limit-state function is expressed as Eq. (1).

1 $$\:G\left(x\right)={R}_{0}-R\left(x\right)$$

Here, *x* refers to the vector of random variables. Reliability analysis then calculates the probability that G(x) ≤ 0, i.e., *EP = P(G(x) ≤ 0)*. The pair ($\:{R}_{0},\:EP)$ constitutes a point on the hazard curve. The value of *R* is determined using a model that depends on the financial risk index of crashes and the design characteristics of the highway.

Throughout the reliability analysis process, the limit-state function is evaluated repeatedly. Several methods have been developed to address reliability problems, including the First-Order Reliability Method (FORM), the Mean Value First-Order Second-Moment Method (MVFOSM), and the Second-Order Reliability Method (SORM), as well as a variety of sampling-based techniques^25^. Among the sampling approaches, Monte Carlo simulation is the most widely applied due to its flexibility and robustness. Given the limitations of analytical methods and the complexity and nonlinearity of limit-state functions, this study adopts Monte Carlo simulation to ensure accurate and reliable results. Reliability analyses were conducted using the *Rt* software, developed by Mahsuli and Haukaas^26^. Additionally, the graphs were generated using *Origin* software.

### Analysis process

In this study, a combined approach was first developed to create a social cost risk index model for crashes. The EB method was used to predict the expected crash frequency and the social cost of crashes for different types of collisions, as proposed by the HSM^27^. Initially, the predicted financial loss for each segment was calculated. Then, utilizing the proposed construction cost values for undivided two-lane highways from the FDOT, a crash risk index model was developed^12^. Equation (2) to (6) illustrate the calculation method for the risk index. Additionally, Tables 1 and 2 present, respectively, the social cost of crashes by collision type and the explanation of symbols used in the study.

2 $$\:{R}_{i}=\frac{{C}_{{expected}_{i}}}{{CC}_{i}}\:\:\:;\:\:\:i=1,\:2,\:3,\:\dots\:,n$$

3 $$\:{C}_{{expected}_{i}}=\sum\:_{j=K}^{O}{C}_{{expected}_{i,j}}\:\:\:;\:\:\:i=1,\:2,\:3,\:\dots\:,n$$

4 $$\:{\:C}_{{expected}_{i,j}}={p}_{j}{N}_{{expected}_{i}}\:\:\:;\:\:\:i=1,\:2,\:3,\:\dots\:,n\:\:\:\:j=K,A,B,C,O$$

5 $$\:{N}_{expected}=w{N}_{predicted}\:+(1-w)\:{N}_{observed}$$

6 $$\:{\:N}_{predicted}=Nspf\:\times\:\:Cr\:\times\:\:{\prod\:}_{1}^{12}{CMF}_{k}\:\:\:;\:\:\:k=1,\:2,\:3,\dots\:,12$$

**Table 1:** Societal crash cost estimates by crash severity^27^.

Collision type	Comprehensive societal crash cost
Fatal (K)	$4,008,900
Disabling injury (A)	$216,000
Evident injury (B)	$79,000
Possible injury (C)	$44,900
PDO (O)	$7,400
Total	$4,356,200

**Table 2:** The explanation of symbols used in the study.

Symbol	Description
$$\:\boldsymbol{G}\left(\boldsymbol{x}\right)$$	The limit-state function
$$\:{\boldsymbol{R}}_{0}$$	The specified threshold of financial risk index
$$\:\boldsymbol{R}\left(\boldsymbol{x}\right)$$	The financial risk index
$$\:{\boldsymbol{R}}_{\boldsymbol{i}}$$	Risk index for segment *i*
$$\:\boldsymbol{x}$$	The vector of random variables
*EP*	The exceedance probability of crash risk index
$$\:{\boldsymbol{C}}_{{\boldsymbol{e}\boldsymbol{x}\boldsymbol{p}\boldsymbol{e}\boldsymbol{c}\boldsymbol{t}\boldsymbol{e}\boldsymbol{d}}_{\boldsymbol{i}}}$$	The social crash cost for segment *i* ($)
$$\:{\boldsymbol{C}\boldsymbol{C}}_{\boldsymbol{i}}$$	The construction cost of segment *i* ($)
$$\:{\boldsymbol{C}}_{{\boldsymbol{e}\boldsymbol{x}\boldsymbol{p}\boldsymbol{e}\boldsymbol{c}\boldsymbol{t}\boldsymbol{e}\boldsymbol{d}}_{\boldsymbol{i},\boldsymbol{j}}}$$	The social crash cost for severity *j* in segment *i* ($)
$$\:{\boldsymbol{p}}_{\boldsymbol{j}}$$	The social crash cost associated with collision severity *j* ($)
$$\:{\boldsymbol{N}}_{{\boldsymbol{e}\boldsymbol{x}\boldsymbol{p}\boldsymbol{e}\boldsymbol{c}\boldsymbol{t}\boldsymbol{e}\boldsymbol{d}}_{\boldsymbol{i}}}$$	The expected crash frequency
$$\:\boldsymbol{w}$$	The weighted adjustment to be placed on the SPF prediction
$$\:{\boldsymbol{N}}_{\boldsymbol{p}\boldsymbol{r}\boldsymbol{e}\boldsymbol{d}\boldsymbol{i}\boldsymbol{c}\boldsymbol{t}\boldsymbol{e}\boldsymbol{d}}$$	Predicted average crash frequency
$$\:{\boldsymbol{N}}_{\boldsymbol{o}\boldsymbol{b}\boldsymbol{s}\boldsymbol{e}\boldsymbol{r}\boldsymbol{v}\boldsymbol{e}\boldsymbol{d}}$$	Observed crash frequency at the site over the study period
$$\:{\boldsymbol{N}}_{\boldsymbol{S}\boldsymbol{P}\boldsymbol{F}}$$	Predicted average crash frequency for base conditions
$$\:{\boldsymbol{C}}_{\boldsymbol{r}}$$	Calibration factor for roadway segments of a specific type or geographical area
$$\:{\boldsymbol{C}\boldsymbol{M}\boldsymbol{F}}_{\boldsymbol{k}}$$	Crash modification factors
$$\:{\boldsymbol{R}}_{\boldsymbol{i},\boldsymbol{c},\boldsymbol{k}}$$	Risk index for segment *i* based on design criterion *c* classification (*k =* Number of subgroups based on criterion c)
$$\:{\boldsymbol{R}}_{\boldsymbol{i},1,\boldsymbol{k}}$$	Risk index for segment *i* based on segment type criterion classification, *k* is equal to 1 for tangent segments and 2 for horizontal curves
$$\:{\boldsymbol{R}}_{\boldsymbol{i},2,\boldsymbol{k}}$$	Risk index for segment *i* based on grade criterion, k is equal to 1 for grade between 0 and 1, 2 for grade between 1 and 2, 3 for grade between 2 and 3, and 4 for grade greater than 3
$$\:{\boldsymbol{R}}_{\boldsymbol{i},3,\boldsymbol{k}}$$	Risk index for segment *i* based on clear zone area criterion, k is equal to 1 for clear zones between 0 and 2 m, 2 for clear zones between 2 and 4 m, and 3 for clear zones greater than 4 m
$$\:{\boldsymbol{R}}_{\boldsymbol{i},4,\boldsymbol{k}}$$	Risk index for segment *i* based on presence or absence of guardrails criterion, k is equal to 1 for the presence of guardrails and 2 for the absence of guardrails
$$\:{\boldsymbol{R}}_{\boldsymbol{i},5,\boldsymbol{k}}$$	Risk index for segment *i* based on lane width criterion, k is equal to 1 for lane width between 3.2 and 3.4, 2 for lane width between 3.4 and 3.6, and 3 for lane width greater than 3.6
$$\:{\boldsymbol{R}}_{\boldsymbol{i},6,\boldsymbol{k}}$$	Risk index for segment *i* based on shoulder type criterion, k is equal to 1 for asphalt shoulder and 2 for gravel shoulder
$$\:{\boldsymbol{R}}_{\boldsymbol{i},7,\boldsymbol{k}}$$	Risk index for segment *i* based on shoulder width criterion, k is equal to 1 for shoulder width between 0 to 0.5 m, 2 for shoulder width between 0.5 to 1 m, and 3 for shoulder width between 1 to 1.5 m
$$\:{\boldsymbol{R}}_{\boldsymbol{i},8,\boldsymbol{k}}$$	Risk index for segment *i* based on RHR criterion, k is equal to 1 for RHR values of 1 and 2, 2 for values of 3 and 4, 3 for values of 5 and 6, and 4 for a value of 7
$$\:{\boldsymbol{R}}_{\boldsymbol{i},9,\boldsymbol{k}}$$	Risk index for segment *i* based on embankment slope criterion, k is equal to 1 for an embankment slope of 01:01, 2 for 01:02, 3 for 01:03, and 4 for slopes greater than 01:04
$$\:{\boldsymbol{R}}_{\boldsymbol{i},10,\boldsymbol{k}}$$	Risk index for segment *i* based on segment length criterion, k is equal to 1 for a length between 0 and 2 km, 2 for a length between 2 and 4 km, and 3 for a length greater than 4 km
$$\:{\boldsymbol{R}\boldsymbol{i}\boldsymbol{s}\boldsymbol{k}}_{\boldsymbol{i}}$$	Risk of segment *i*

Using the calculated risk index values for each segment, a reliability analysis was conducted to compute the exceedance probability based on highway design criteria. The hazard curve was plotted for all road design criteria. These graphs illustrate the distribution of the risk index for each segment type across different values of the selected criterion. Additionally, a reference hazard curve was drawn for all segments, which can be used for prioritizing the segments. Finally, the risk value for each segment was computed using Eq. (7).

7 $$\:{Risk}_{i}={EP}_{i}\times\:{R}_{i}$$

The prioritization of segments for safety improvements is carried out using the values calculated from Eq. (7).

### Data collection

The dataset used in this research pertains to Highway No. 36, a 186-kilometer segment linking the cities of Shadmehr, Kashmar, Khalilabad, Bardaskan, and Sabzevar in Iran’s Razavi Khorasan Province. This two-lane rural highway consists of 34 tangent segments and 30 curved segments. The collected data are classified into three main categories: geometric characteristics, traffic information, and crash records for the years 2019 to 2023.

Geometric data were obtained through on-site surveys and verified against the digital road inventory maintained by the Khorasan Razavi Road Maintenance and Transportation Organization. Key parameters such as lane width, shoulder width and type, embankment slope, and curve radius were extracted and digitized using AutoCAD software.

Traffic and crash data were retrieved from the official database of the Khorasan Razavi Road Maintenance and Transportation Organization. The traffic dataset included average annual daily traffic (AADT) and vehicle composition for each segment, derived from automatic counting stations. The crash dataset contained detailed records of crash frequency, severity, and location, which were spatially matched to the corresponding highway segments.

Data processing and quality control involved cross-checking geometric parameters with field inspection reports, validating traffic volumes against annual count summaries, and removing crash records with incomplete or inconsistent information. All variables were standardized and converted to consistent units before being merged into a unified dataset used for subsequent analysis.

### Limit-state functions

Based on Eq. (1), the function $\:{R}_{0}$ in this study is defined as the risk index of each segment for the corresponding roadway category. The value $\:R\left(x\right)$ represents different levels of risk, which are considered to examine variations in the risk index. Within the reliability framework, the solution essentially indicates the probability of the risk index exceeding these predefined levels, thereby enabling the assessment of safety and the prioritization of projects. The general form of the limit-state function is given by Eq. (8).

8 $$\:G\left(x\right)={R}_{0}-{R}_{i,c,k}\:;\:\:\:i=\mathrm{1,2},3,\dots\:n\:\:\:\:\:\:\:c=\mathrm{1,2},3,\dots\:,10\:\:\:\:\:\:\:\:k=Number\:of\:subgroups\:based\:on\:criterion\:c\:$$

In which *n* represents the number of segments based on the values of each criterion, and the values of *c* ranging from 1 to 10 correspond to the criteria of segment type, grade, clear zone, presence or absence of side guardrails, lane width, shoulder type, shoulder width, roadside hazard rating (RHR), embankment slope, and segment length.

## Results

For reliability analysis, it is first necessary to determine the statistical characteristics and the probability distribution type of the random variables. These characteristics influence the formation of the joint probability density function (PDF) of the limit-state functions. The results of the Kolmogorov-Smirnov test, used to determine the probability distribution and statistical properties of the random variables, are presented in (Table 3).

**Table 3:** Descriptive statistics and Kolmogorov-Smirnov test results.

Variable	*N* total	Mean	S.D.	Min	Max	Distribution
$\:{\boldsymbol{R}}_{\boldsymbol{i},1,1}$	34	4.86	4.03	1.02	17.86	Gamma
$\:{\boldsymbol{R}}_{\boldsymbol{i},1,2}$	30	2.64	1.85	0.07	7.04	Gamma
$\:{\boldsymbol{R}}_{\boldsymbol{i},2,1}$	18	3.84	3.39	0.87	13.18	Gamma
$\:{\boldsymbol{R}}_{\boldsymbol{i},2,2}$	20	2.26	2.42	0.06	10.06	Gamma
$\:{\boldsymbol{R}}_{\boldsymbol{i},2,3}$	16	1.88	1.90	0.05	5.91	Gamma
$\:{\boldsymbol{R}}_{\boldsymbol{i},2,4}$	10	2.11	1.72	0.18	5.02	Gamma
$\:{\boldsymbol{R}}_{\boldsymbol{i},3,1}$	34	2.37	2.60	0.05	13.18	Gamma
$\:{\boldsymbol{R}}_{\boldsymbol{i},3,2}$	14	2.16	1.54	0.36	4.81	Gamma
$\:{\boldsymbol{R}}_{\boldsymbol{i},3,3}$	16	3.42	3.25	0.72	11.58	Gamma
$\:{\boldsymbol{R}}_{\boldsymbol{i},4,1}$	15	2.67	3.30	0.40	13.18	Gamma
$\:{\boldsymbol{R}}_{\boldsymbol{i},4,2}$	49	2.56	2.40	0.05	11.58	Gamma
$\:{\boldsymbol{R}}_{\boldsymbol{i},5,1}$	25	1.85	1.62	0.05	5.91	Gamma
$\:{\boldsymbol{R}}_{\boldsymbol{i},5,2}$	18	2.08	1.71	0.27	5.79	Gamma
$\:{\boldsymbol{R}}_{\boldsymbol{i},5,3}$	21	3.89	3.61	0.63	13.18	Gamma
$\:{\boldsymbol{R}}_{\boldsymbol{i},6,1}$	37	3.15	3.01	0.36	13.18	Gamma
$\:{\boldsymbol{R}}_{\boldsymbol{i},6,2}$	27	1.82	1.68	0.05	5.79	Gamma
$\:{\boldsymbol{R}}_{\boldsymbol{i},7,1}$	27	1.90	1.72	0.05	5.91	Gamma
$\:{\boldsymbol{R}}_{\boldsymbol{i},7,2}$	11	2.49	2.02	0.18	6.21	Gamma
$\:{\boldsymbol{R}}_{\boldsymbol{i},7,3}$	26	3.34	3.36	0.36	13.18	Gamma
$\:{\boldsymbol{R}}_{\boldsymbol{i},8,1}$	9	3.27	3.53	0.87	11.58	Gamma
$\:{\boldsymbol{R}}_{\boldsymbol{i},8,2}$	7	4.00	4.25	0.72	13.18	Gamma
$\:{\boldsymbol{R}}_{\boldsymbol{i},8,3}$	20	2.68	2.43	0.36	10.06	Gamma
$\:{\boldsymbol{R}}_{\boldsymbol{i},8,4}$	28	1.95	1.70	0.05	5.79	Gamma
$\:{\boldsymbol{R}}_{\boldsymbol{i},9,1}$	28	1.95	1.70	0.05	5.79	Gamma
$\:{\boldsymbol{R}}_{\boldsymbol{i},9,2}$	10	3.84	2.83	0.90	10.06	Gamma
$\:{\boldsymbol{R}}_{\boldsymbol{i},9,3}$	13	1.73	1.11	0.63	4.43	Gamma
$\:{\boldsymbol{R}}_{\boldsymbol{i},9,4}$	13	3.85	4.14	0.36	13.18	Gamma
$\:{\boldsymbol{R}}_{\boldsymbol{i},10,1}$	32	3.80	3.14	0.06	13.18	Gamma
$\:{\boldsymbol{R}}_{\boldsymbol{i},10,2}$	18	1.46	1.13	0.05	3.766	Gamma
$\:{\boldsymbol{R}}_{\boldsymbol{i},10,3}$	14	1.25	0.70	0.18	2.71	Gamma
$\:{\boldsymbol{R}}_{\boldsymbol{i}}$	64	8.93	10.82	0.08	72.61	Lognormal

Table 3 indicates that most random variables in the reliability analysis, including segment *i* risk index, follow a Gamma distribution, confirmed by the Kolmogorov–Smirnov test. In addition to the Kolmogorov–Smirnov test, graphical goodness-of-fit checks were also applied to validate the selection of distributions. The crash risk index ($\:{R}_{i}$), however, exhibits a Lognormal distribution, which is consistent with the nature of composite risk measures that result from the product of multiple independent positive variables. The mean and standard deviation values reflect considerable variability across segments. In several variables, the mean exceeds the standard deviation, which is typical of positively skewed distributions and indicates long-tailed behavior. This statistical variability reinforces the importance of applying a probabilistic framework, as randomness in input variables can significantly affect the reliability estimates of crash risk. The observed variance differences are mainly due to heterogeneity in roadway geometric features. These characteristics cause small design variations to nonlinearly affect risk, thereby increasing variance across groups.

### Risk index analysis

**Fig. 1:**
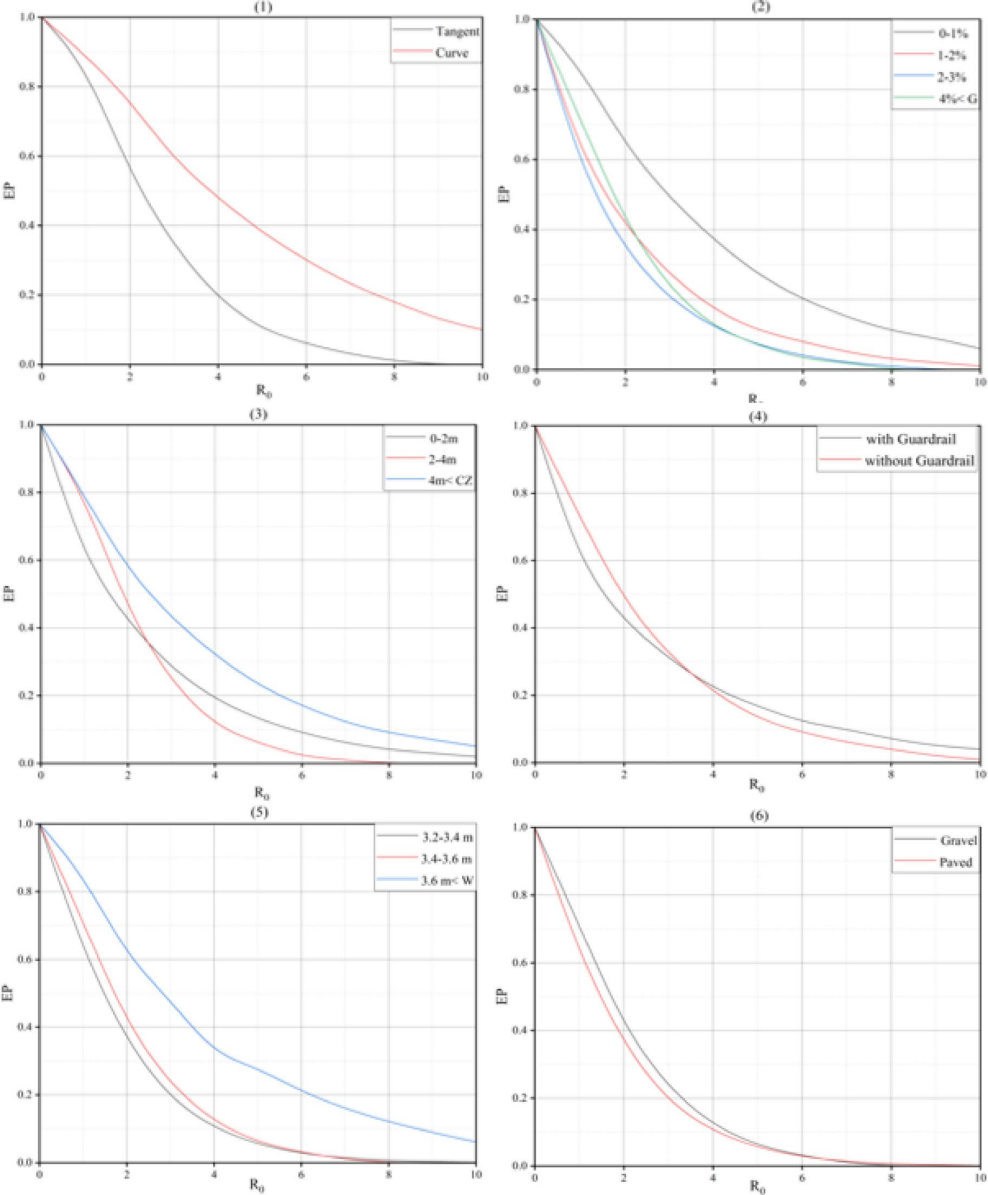

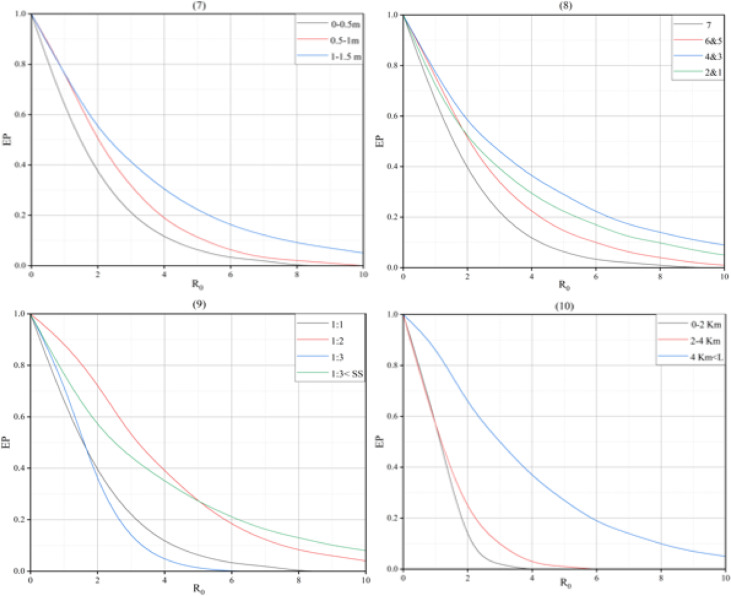
presents reliability analysis results, showing the risk index exceedance probability beyond the tolerable threshold per design criteria.

Figure 1 Exceedance probability graphs for (1) segment type, (2) grade, (3) clear zone, (4) guardrail, (5) lane width, (6) shoulder type, (7) shoulder width, (8) RHR, (9) side slope and (10) segment length.

The results obtained from the analysis of the segment type criterion graph indicate that the risk index is higher in horizontal curve segments compared to tangent segments. The graph related to the grade criterion shows that as the grade increases from 0 to 3%, the risk index decreases. However, this pattern deviates for grades greater than 4%. The graph related to the clear zone criterion shows that for lower values of the risk index (up to 2.5), the general trend indicates an increase in the risk index as the clear zone increases. However, for higher values of the risk index, this pattern does not hold for clear zone between 0 and 4 m. The results indicate guardrails reduce risk for index values below 3.5, but increase risk when the index exceeds 3.5. Risk plots reveal that guardrail-containing segments frequently coincide with other adverse geometric or roadside conditions at elevated risk index levels. These combined characteristics can contribute to the elevated risk values observed in such segments. This pattern indicates that elevated risk is not solely due to guardrails but reflects the broader roadway environment context. The results for the lane width criterion indicate that as the lane width increases, the risk index also increases. Notably, this increase is more significant when the lane width exceeds 3.6 m compared to changes in the other two categories. The analysis of the shoulder type criterion showed that the risk index is higher in gravel shoulders compared to asphalt shoulders for values of the risk index lower than 6.5. Additionally, the graph for the shoulder width criterion revealed that as the shoulder width increases, the risk index increases in most intervals. Risk variations with lane and shoulder width may result from extra maneuvering space, increasing perceived safety and encouraging higher speeds. Under such conditions, the severity of crashes is more likely to increase when a collision occurs, even if the overall crash frequency remains unchanged. This pattern suggests that the elevated risk index reflects a shift in crash severity rather than an increase in crash occurrence. The graph for the RHR index did not follow a clear pattern. Specifically, as the values move from 3 to 4, 1 and 2, 6 and 5, to 7, the risk index decreases. Finally, the graph for the segment length criterion showed that as the segment length increases, the risk index decreases. Notably, the rate of risk reduction between the length range of 0 to 2 km and 2 to 4 km is more significant than for other changes.

### Comparison of design criteria

**Fig. 2:**
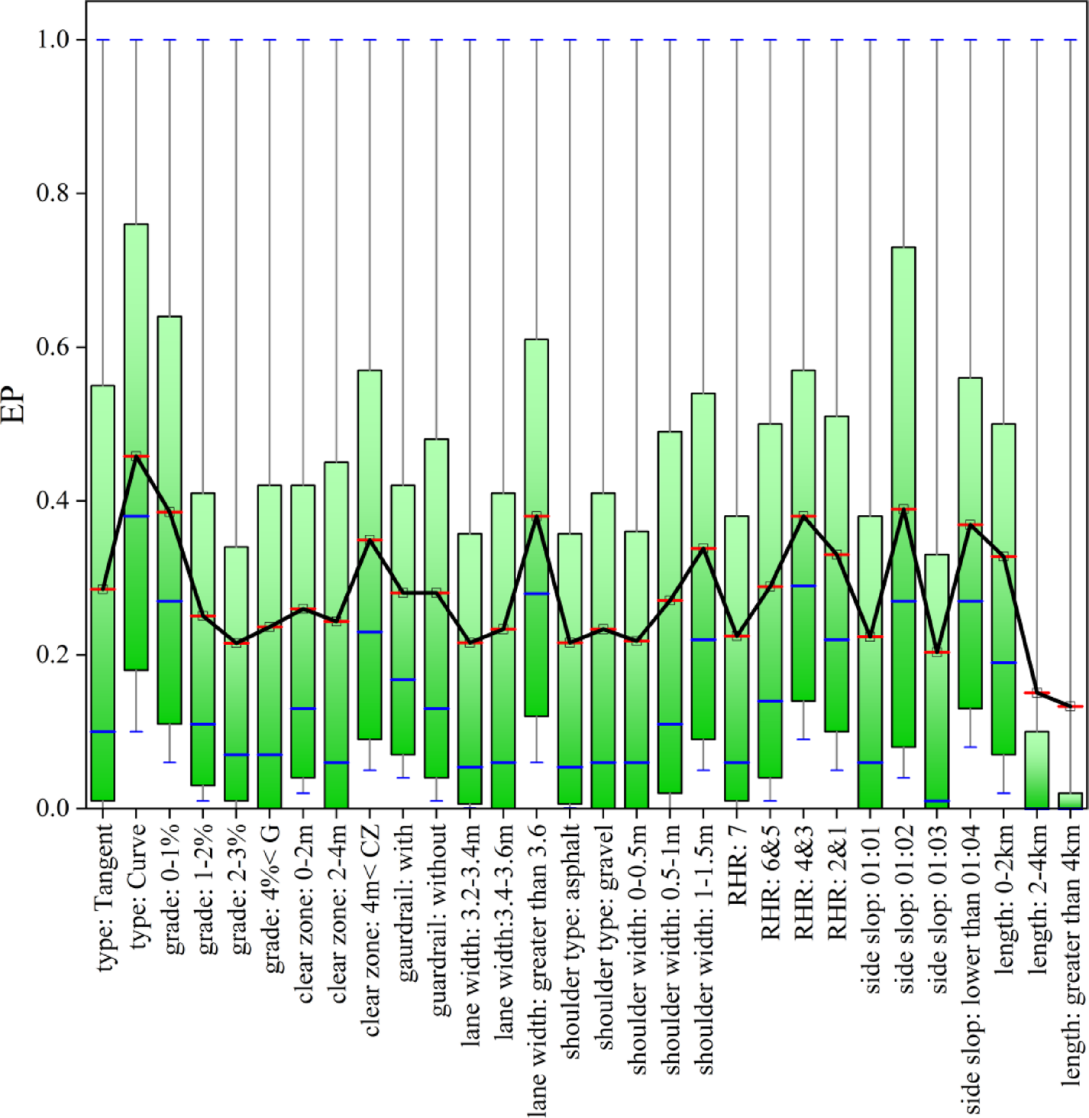
Box plot of risk index across highway design criteria.

Based on the highway specifications criteria, the statistical parameters of the risk index for each criterion are presented in (Fig. 2).

In this box plot, the dispersion of the risk index for each criterion within the 25th to 75th percentile range is represented by the box. The vertical lines outside the box connect to the minimum and maximum values. The blue dashed lines represent the median values, while the red dashed lines indicate the mean values of the risk index. The plot shows the highest risk index for horizontal curves and the lowest for segments longer than 4 km.

### Project prioritization

**Fig. 3:**
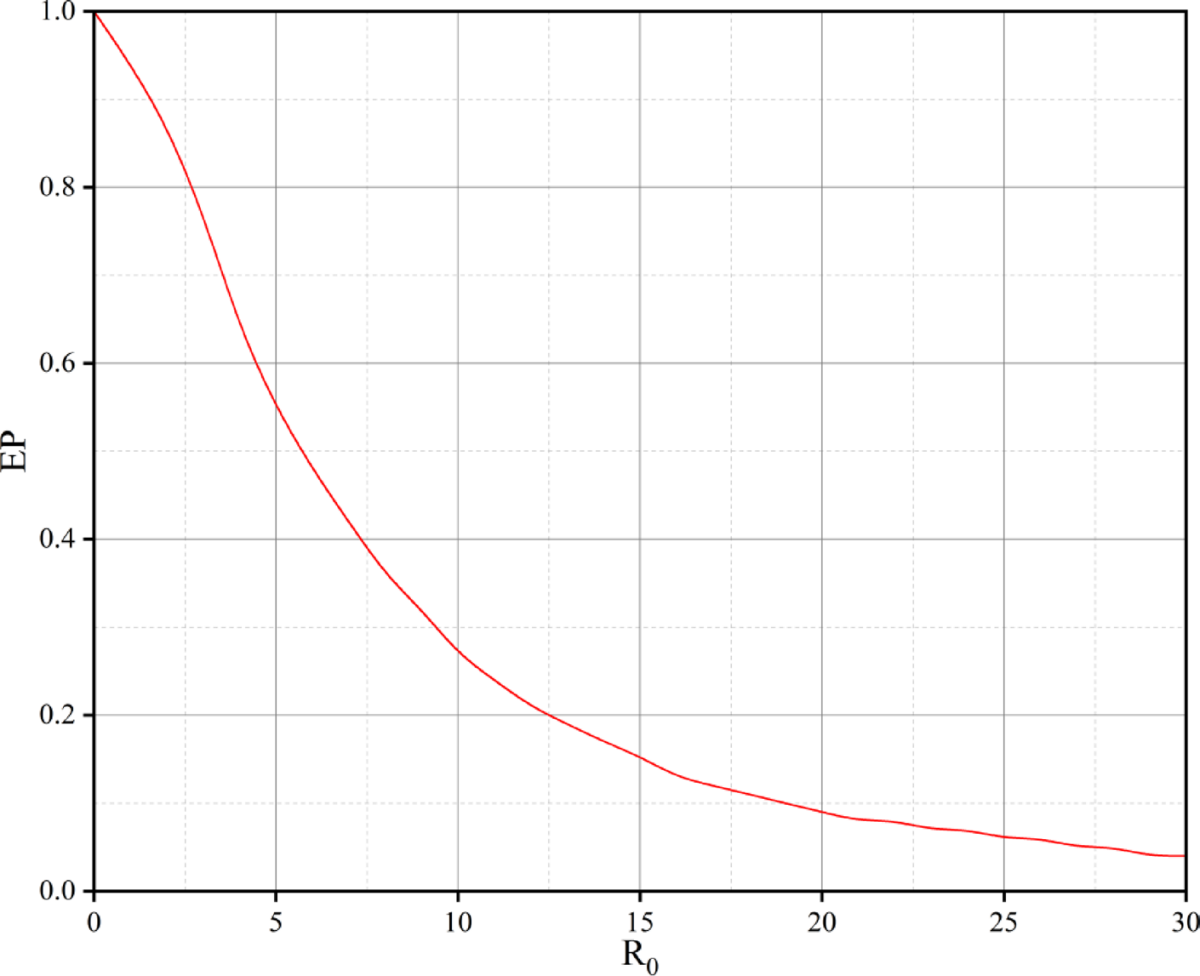
Reference risk curve.

To prioritize the safety enhancement projects, the probability of exceedance of the risk index for all segments has been analyzed within a limit-state function. Figure 3 illustrates the probability of exceedance of the risk index for all segments, without considering the specific design criteria of the highway.

In this figure, the product of the risk index values and the probability of exceedance represents the risk magnitude for each segment. This figure, referred to as the “reference risk curve,” is utilized for prioritizing safety enhancement projects. Table 4 presents the project prioritization results. To prioritize safety improvement projects, the final risk value for each segment was computed by multiplying the socio-economic risk index ($\:{R}_{i}$) by its corresponding exceedance probability (*EP*). This product, shown in the third column of Table 4, reflects the relative importance of intervention at each segment. In this context, the term “project” refers to each individual road segment, treated as a standalone unit for safety decision-making purposes. Table 4 presents three columns: (1) the socio-economic risk index ($\:{R}_{i}$), (2) the probability of exceedance (*EP*), and (3) the resulting risk value used for prioritization. These values are ranked in descending order to support efficient resource allocation toward segments posing the highest crash risks.

**Table 4:** Safety enhancement project prioritization.

No segment	$\:{\boldsymbol{R}}_{\boldsymbol{i}}$	EP	$\:{\boldsymbol{R}\boldsymbol{i}\boldsymbol{s}\boldsymbol{k}}_{\boldsymbol{i}}$	$\:{\boldsymbol{R}\boldsymbol{a}\boldsymbol{n}\boldsymbol{k}}_{\boldsymbol{R}}$
1	19.922	0.095	1.893	48
2	7.543	0.390	2.942	2
3	19.657	0.097	1.907	47
4	2.531	0.839	2.123	44
5	1.287	0.921	1.185	57
6	5.041	0.548	2.763	15
7	5.084	0.541	2.751	16
8	11.458	0.231	2.647	23
9	9.984	0.278	2.775	11
10	2.669	0.811	2.165	35
11	11.439	0.232	2.654	22
12	10.033	0.277	2.779	9
13	5.043	0.548	2.764	14
14	17.439	0.118	2.058	45
15	12.547	0.202	2.534	28
16	5.105	0.538	2.747	19
17	12.646	0.200	2.529	29
18	2.539	0.838	2.128	41
19	5.074	0.546	2.770	12
20	7.614	0.386	2.939	3
21	10.054	0.277	2.785	7
22	2.538	0.838	2.127	42
23	16.373	0.132	2.161	36
24	2.537	0.838	2.126	43
25	10.072	0.276	2.780	8
26	5.080	0.541	2.748	18
27	1.273	0.921	1.173	61
28	1.283	0.921	1.182	60
29	1.285	0.921	1.184	59
30	2.554	0.836	2.135	39
31	5.076	0.541	2.746	21
32	18.830	0.104	1.958	46
33	1.285	0.921	1.184	58
34	6.325	0.461	2.916	6
35	5.077	0.541	2.746	20
36	1.313	0.921	1.209	54
37	3.131	0.767	2.402	31
38	1.294	0.921	1.192	56
39	10.033	0.277	2.779	9
40	7.602	0.386	2.934	4
41	2.580	0.834	2.152	38
42	2.964	0.774	2.295	33
43	2.547	0.836	2.130	40
44	1.300	0.921	1.198	55
45	2.931	0.780	2.286	34
46	0.086	1.000	0.086	64
47	0.249	0.999	0.249	62
48	5.167	0.532	2.749	17
49	12.789	0.196	2.507	30
50	5.323	0.520	2.768	13
51	7.868	0.372	2.927	5
52	4.279	0.608	2.602	24
53	14.727	0.159	2.342	32
54	4.063	0.631	2.564	25
55	72.617	0.003	0.218	63
56	29.859	0.041	1.224	53
57	20.087	0.093	1.868	49
58	23.415	0.068	1.592	50
59	28.628	0.044	1.260	52
60	3.804	0.669	2.545	26
61	25.011	0.059	1.476	51
62	2.581	0.834	2.153	37
63	3.806	0.668	2.542	27
64	7.568	0.389	2.944	1

The resulting prioritization allows decision-makers to focus on the segments that present the highest combined risk level in both severity and probability terms. A key contribution of the proposed framework is its ability to combine roadway geometric and traffic characteristics with severity-based social crash costs and construction costs within a single evaluation structure. Linking roadway conditions to crash outcomes and economic impacts provides a coherent basis for prioritizing safety improvements. This probabilistic approach lets agencies align decisions with risk thresholds, offering flexibility and analytical depth beyond traditional methods.

## Discussion

This study provides new perspectives on how highway geometric design parameters influence crash risk and how probabilistic modeling can improve decision-making in transportation safety. The results generally align with previous studies, particularly in highlighting the elevated risk associated with horizontal curves. For instance, the finding that crash risk increases on curves due to centrifugal force and reduced visibility supports earlier work by Himes and Donnell^19^ and Shalkamy and El-Basyouny^18^. Similarly, the reduced risk associated with longer segments echoes the conclusions of You et al.^6^, which emphasized the stabilizing effects of uninterrupted roadway design.

The effect of grade follows a nonlinear pattern. Risk decreases with grade between 0% and 3%, aligning with findings that gentle grades enhance vehicle stability^17^. However, slopes greater than 4% elevate crash risk due to increased speed variability and longer braking distances. Similarly, the relationship between clear zone width and crash risk varies at different levels. For lower risk values (up to 2.5), a wider clear zone is associated with a higher crash risk, likely due to driver overconfidence. However, at higher risk levels, this trend becomes less pronounced for clear zones between 0 and 4 m.

The presence of guardrails has a dual effect. When the risk index is below 3.5, guardrails help reduce crash risk. However, at higher risk levels, drivers may engage in riskier behaviors due to perceived safety, potentially increasing crash risk. Additionally, crash risk tends to rise as lane width increases, particularly for widths exceeding 3.6 m, likely because wider lanes encourage higher vehicle speeds. Gravel shoulders are linked to a higher crash risk than asphalt shoulders, underscoring the importance of proper maintenance. Likewise, wider shoulders may unintentionally promote erratic driving behavior, further elevating crash risk.

The RHR analysis does not exhibit a consistent pattern, likely due to the complex interplay of multiple influencing factors. Meanwhile, segment length plays a crucial role in crash risk reduction. Longer segments generally correspond to lower crash risk, with the most substantial reduction occurring when segment length increases from 0 to 2 km to 2–4 km. This trend suggests that frequent interruptions, such as intersections, may contribute to higher crash rates.

This study enhances highway safety research by integrating reliability analysis with a socio-economic risk index to prioritize safety improvements. Unlike previous studies that primarily relied on historical crash data, this approach incorporates a probabilistic framework to account for uncertainty in risk assessment.

From a practical perspective, the study introduces a socio-economic crash risk index that considers both crash severity and construction cost. This enables transportation agencies to prioritize investments based on both safety impact and cost-effectiveness. The results are especially relevant for policymakers and project planners who must allocate limited resources while maximizing social benefit.

Beyond its regional focus, the integrated socio-economic and reliability-based approach proposed in this study establishes a transferable analytical framework that can be adapted to diverse highway environments. This methodological advancement contributes to the global discourse on risk-informed infrastructure management, where probabilistic modeling and cost-based prioritization are increasingly recognized as essential tools for evidence-based safety policy.

Future research should investigate the interactions between different design parameters, as this study evaluated each factor independently. Incorporating real-time traffic and driver behavior data could improve predictive accuracy and offer deeper insights into dynamic crash risk factors. By refining these methodologies, transportation agencies can implement more targeted and efficient strategies for mitigating crash risks and improving highway safety.

A primary limitation is the reliance on US-specific models and cost data, including the HSM empirical Bayes framework and FDOT construction estimates. These sources ensured methodological consistency, yet economic, institutional, and contextual differences—e.g., U.S. vs. Iran—may limit direct generalizability. Additionally, since the cost-related data are drawn from a single national context, future research could benefit from incorporating multi-country datasets to enhance cross-contextual robustness and precision. Nevertheless, this study aims to provide a methodological framework for relative risk assessment rather than to derive absolute values, which helps mitigate the implications of this limitation. Future work should consider integrating localized models and cost estimates to strengthen the contextual validity and practical applicability of the results. Nevertheless, the conceptual integration of socio-economic valuation with reliability analysis represents a substantial step forward in the evolution of quantitative highway safety modeling. It provides a replicable foundation for future cross-country studies seeking to align infrastructure investment decisions with measurable risk reduction outcomes.

## Conclusion

This study shows horizontal curves, wider lanes, gravel shoulders, and insufficient clear zones significantly increase highway crash risk. Conversely, longer segments and controlled slopes help reduce risk. The use of a reliability-based probabilistic approach offers an effective method for prioritizing road safety improvements.

A significant contribution of this study is the development of a socio-economic risk index, which considers both crash costs and construction expenses. This integrated approach enables transportation agencies to optimize resource allocation and make data-driven, cost-effective safety decisions. The framework developed here extends beyond Iran, offering a systematic, scalable approach for evaluating safety risks in other developing regions.

Despite these contributions, the study is based on historical crash data and geometric features, without incorporating real-time traffic flow or driver behavior dynamics. Future research should address this limitation by incorporating real-time data and advanced modeling techniques to further refine risk predictions. Ultimately, these findings provide valuable insights for policymakers and engineers, helping them implement more effective strategies to reduce crashes and improve highway safety on a broader scale.

Beyond the specific findings, the research contributes a comprehensive and practical framework for probabilistic risk modeling and reliability-based analysis in roadway safety. By integrating crash data, roadway characteristics, and probabilistic distributions of risk indices, the approach effectively captures both cognitive and inherent uncertainties as well as the interdependencies among variables. This reliability-based framework offers a robust and adaptable tool for prioritizing safety interventions and evaluating trade-offs between societal crash costs and construction costs. Moreover, the methodology is versatile and can be extended to other crash cost models and construction cost coefficients across different jurisdictions, making it a valuable resource for both researchers and policymakers.

## Data Availability

The datasets used and/or analysed during the current study available from the corresponding author on reasonable request.
